# Composition of subgingival microbiota associated with periodontitis and diagnosis of malignancy—a cross-sectional study

**DOI:** 10.3389/fmicb.2023.1172340

**Published:** 2023-05-22

**Authors:** Aswathy Narayanan, Birgitta Söder, Jukka Meurman, Anna Lundmark, Yue O. O. Hu, Ujjwal Neogi, Tülay Yucel-Lindberg

**Affiliations:** ^1^Division of Clinical Microbiology, Department of Laboratory Medicine, ANA Futura, Karolinska Institutet, Stockholm, Sweden; ^2^Division of Infectious Diseases, Department of Medicine Huddinge, Karolinska Institutet, Stockholm, Sweden; ^3^Division of Periodontology, Department of Dental Medicine, Karolinska Institutet, Huddinge, Sweden; ^4^Department of Oral and Maxillofacial Diseases, University of Helsinki and Helsinki University Hospital, Helsinki, Finland; ^5^Division of Pediatric Dentistry, Department of Dental Medicine, Karolinska Institutet, Huddinge, Sweden; ^6^Department of Microbiology, Tumor and Cell Biology, Centre for Translational Microbiome Research, Karolinska Institutet, Stockholm, Sweden; ^7^School of Environmental Science and Engineering, Hubei Polytechnic University, Huangshi, China; ^8^The Systems Virology Lab, Division of Clinical Microbiology, Department of Laboratory Medicine, ANA Futura, Karolinska Institutet, Stockholm, Sweden

**Keywords:** periodontitis, supragingival plaque, cancer, malignancy, 16S rRNA gene sequencing, oral microbiota

## Abstract

Periodontitis is one of the world’s most prevalent infectious conditions, affecting between 25 and 40% of the adult population. It is a consequence of the complex interactions between periodontal pathogens and their products, which trigger the host inflammatory response, chronic inflammation, and tissue destruction. Chronic systemic low-grade inflammation is involved in numerous diseases, and it is also known that long-lasting inflammation and chronic infections predispose one to cancer. Here, we characterized and compared the subgingival microbiota associated with periodontitis and diagnosis of malignancy in a longitudinal 10-year follow-up study. The study was conducted on 50 patients with periodontitis and 40 periodontally healthy individuals. The recorded clinical oral health parameters were periodontal attachment loss (AL), bleeding on probing (BOP), gingival index (GI), probing depth (PD), and plaque index (PI). Subgingival plaque was collected from each participant, from which DNA was extracted, and 16S rRNA gene amplicon sequencing performed. Cancer diagnoses data were collected between the years 2008–2018 from the Swedish Cancer Registry. The participants were categorized based on having cancer at the time of sample collection (CSC), having developed cancer later (DCL), and controls without any cancer. The most abundant phyla across all 90 samples were *Actinobacteria*, *Proteobacteria*, *Firmicutes*, *Bacteroidetes*, and *Fusobacteria*. At the genus level, *Treponema*, *Fretibacterium*, and *Prevotella* were significantly more abundant in samples of periodontitis patients compared to non-periodontitis individuals. With regard to samples of cancer patients, *Corynebacterium* and *Streptococcus* were more abundant in the CSC group; *Prevotella* were more abundant in the DCL group; and *Rothia*, *Neisseria*, and *Capnocytophaga* were more abundant in the control group. In the CSC group, we also found that the presence of periodontal inflammation, in terms of BOP, GI, and PLI, significantly correlated with species belonging to the genera *Prevotella*, *Treponema*, and *Mycoplasma*. Our results revealed that several subgingival genera were differentially enriched among the studied groups. These findings underscore the need for further research to fully understand the role that oral pathogens may play in the development of cancer.

## Introduction

The oral cavity harbors thousands of different microbial species that can be found on soft tissue and teeth forming biofilms, or communities of microorganisms attached to a surface (Keijser et al., 2008). The most prevalent oral biofilm, dental plaque, exists on tooth surfaces in the form of complex multispecies communities. As the biofilm matures and develops, there is also a gradual shift from Gram-positive aerobic bacteria towards Gram-negative and anaerobic species, affecting the gingival environment with respect to pH and oxygen levels, which promotes species favored by this milieu (Asikainen and Chen, 1999; O'Toole et al., 2000). In addition, the inflammatory response from the host can enrich the environment with inflammatory mediators that enhance the growth of certain “inflammophilic” bacteria, which feed off inflammatory products (Hajishengallis, 2014). Such inflammation is generally resolved in normal healing processes, whereas insufficient resolution results in neutrophil-mediated chronic inflammation and destruction of tissue and bone structures (Serhan, 2014). Chronic inflammation involves several diseases, such as rheumatoid arthritis, periodontitis, type 2 diabetes mellitus, and cardiovascular disease. It is also known that long-lasting inflammation, secondary to chronic infections or infectious agents, predisposes one to cancer development (Coussens and Werb, 2002; de Martel et al., 2012; Garrett, 2015).

Periodontal disease (periodontitis) is a major cause of tooth loss in adults and one of the world’s most prevalent chronic infectious inflammatory diseases, affecting up to 25–40% of the adult population. The most severe form of the disease affects 5–15% of the global population (Page and Eke, 2007; Dye, 2012; Eke et al., 2015). Periodontitis is characterized by the destruction of tooth-supporting tissue and bone, which may ultimately result in tooth loss. The disease results from the complex interactions between periodontal microorganisms and their products, triggering the host inflammatory response. The process is initiated when a biofilm forms near the gingiva and releases various substances, such as lipopolysaccharides, peptidoglycans, and toxins, which elicit a host response (Page and Kornman, 1997; Pollanen et al., 2012; Yucel-Lindberg and Bage, 2013). The “red complex” bacteria comprising *Porphyromonas gingivalis (P. gingivalis)*, *Treponema denticola*, and *Tannerella forsythia* has long been associated with the disease, but this view has changed with the emergence of new technologies towards a model where periodontitis is associated with a shift in the whole microbial composition rather than focusing on individual microbial species. As a consequence of bacterial challenge, the host immune response initiates the activation and stimulation of pro-inflammatory cytokines, chemokines, prostaglandins, toll-like receptors, and proteolytic enzymes, collectively contributing to the pathogenesis of periodontitis (Bascones et al., 2005). The expression and/or production of these factors have been demonstrated using gingival tissue biopsies, gingival fluid, and saliva, as well as different types of oral cells (Båge et al., 2011; Davanian et al., 2012; Cavalla et al., 2015). The ongoing “battle” of inflammation is not only measurable locally in the oral samples but also systemically, as increased levels of inflammatory mediators have been demonstrated in the blood of patients with oral diseases, particularly in those with periodontitis (Van Dyke, 2009; Hajishengallis and Chavakis, 2021).

Chronic inflammatory conditions associated with infections may lead to environments that promote genomic lesions and the initiation of tumors. Previous studies have reported an association between periodontitis and an increased risk of total cancer (Romandini et al., 2021; Kim et al., 2022). One meta-regression analysis based on seven case–control studies showed a statistically higher risk of oral cancer with increasing number of missing teeth, with the latter considered a proxy for chronic dental/oral infections (Virtanen et al., 2014). A systematic review and meta-analysis performed recently demonstrated that periodontal disease significantly increases the risk of colorectal cancer by 44% (Li et al., 2021). Additionally, it has been reported that oral squamous cell carcinoma, representing 95% of oral malignancies, is associated with alterations in the oral microbiome. Several studies have linked oral microbiota and periodontal pathogens to head and neck cancer, pancreatic cancers, and colorectal cancer (Ahn et al., 2012; Michaud, 2013; Flemer et al., 2018; Irfan et al., 2020). For example, both *in vivo* and *in vitro* studies have suggested that the key periodontal pathogen *P. gingivalis* contributes to oral carcinogenesis (Groeger et al., 2011; Gallimidi et al., 2015). In contrast, it was reported (Soder et al., 2021) that *P. gingivalis* and *Prevotella intermedia* were more prevalent among subjects without malignancy, whereas the periodontal bacteria *Aggregatibacter actinomycetemcomitans* was strongly associated with malignancy. According to different meta-analyses and reviews (Michaud et al., 2017; Nwizu et al., 2017; Li et al., 2022), the existing data provide support for an association between periodontal disease and risk of different types of cancer including head and neck, lung, colorectal, and pancreatic cancers, although additional research efforts are necessary to further identify the role of oral infections in malignancy. In this study, we aimed to characterize and compare subgingival microbiota associated with periodontitis and the diagnosis of cancer, data extracted from national register of malignancies, in a longitudinal 10-year follow-up study, using 16S rRNA gene sequence analysis.

## Materials and methods

### Sample collection, DNA extraction and sequencing

A total of 99 individuals divided into two groups, a periodontitis group (*n* = 55) and a non-periodontitis control group (*n* = 44), were included in the present study. The participants of this study were derived from our Swedish cohort study, which was described in detail previously (Soder et al., 2007). In total, 1,676 participants (838 women and 838 men) were randomly selected from a database registry of all citizens of Stockholm County who were born on the 20th of the month (between years 1945–1954) and underwent an initial oral clinical examination (1985). In 2009, the participants were clinically reexamined for the prevalence of periodontal disease, from which 99 age- and gender-matched subjects with and without periodontitis were enrolled in the study. The included oral clinical parameters were gingival index (GI), pocket depth (PD), bleeding on probing (BOP), clinical attachment loss (CAL), and plaque index (PLI). For each tooth, BOP and CAL were assessed from six different surfaces using a periodontal probe (HU-FRIEDY Perio Probe). The criteria used for the classification of periodontitis were at least one site with PD ≥5 mm, CAL ≥5 mm, and BOP as described previously (Soder et al., 2007; Yakob et al., 2012). Subgingival plaque samples were carefully collected from four sites on each participant, from the second premolar in each quadrant, and stored at −80°C until microbiome analysis.

The diagnoses of malignancy were obtained from the Swedish Cancer Registry included in the registers of the National Board of Health and Welfare, Sweden. For the present study, the 10-year cumulative cancer diagnoses were collected between the years 2009–2018. The cancer cases that had been diagnosed were: orodigestive cancer, breast cancer, prostate cancer, gynaecological cancers, haematological malignancies, head and neck cancers and liver cancer. The study was approved by the Ethics Committee of the Karolinska University Hospital at Huddinge (Dnr 2007/1669-31; 2012/590-32; 2017/2204-32), and all participants gave their informed consent to be included in the study.

### DNA extraction, 16S rRNA gene amplification, and sequencing

DNA was extracted from the 99 subgingival plaque samples (pooled together from all sites) using the QIAamp DNA Mini Kit (Qiagen, Valencia, CA, United States) and eluted into 50 μL H_2_O. The V3–V4 regions of the bacterial 16S rRNA gene were amplified with 1.0 μM 341′F primer (CCTAHGGGRBGCAGCAG), 1.0 μM 805R primer (GACTACHVGGGTATCTAATCC) (Herlemann et al., 2011), KAPA HotStart ReadyMix (Biosystems, Wilmington, MA, United States), 0.5 ng/μL bovine serum albumin (New England Biolabs, Ipswich, MA, United States), and 2.0 ng of DNA. PCR was performed at 98°C for 2 min followed by 26 cycles of 98°C for 20 s, 54°C for 20 s, and 72°C for 15 s, and a final elongation step of 72°C for 2 min. The samples were purified with polyethylene glycol 6000 (Merck Millipore, Darmstadt, Germany) and carboxylic acid beads (Dynabeads^®^ MyOne^™^, Thermo Fisher Scientific, Waltham, MA, United States) using the procedure described by Lundin et al. (2010). Thereafter, 12 μL of the amplified and purified product was used for indexing (0.4 μM forward and 0.4 μM reverse indexing primer and KAPA HotStart ReadyMix). The conditions for PCR cycling were 98°C for 2 min followed by 10 cycles of 98°C for 20 s, 62°C for 30 s, and 72°C for 30 s, and a final step of 72°C for 2 min. After amplification, the samples were quantified using a Qubit® 2.0 Fluorometer (Invitrogen, Carlsbad, CA, United States), diluted to 2.0 ng/μL, and pooled before purification by the same procedure as described above. An Agilent 2100 Bioanalyzer (Agilent Technologies, Santa Clara, CA, United States) and a Qubit^®^ 2.0 Fluorometer (Invitrogen, Carlsbad, CA, United States) were used for checking the amplicon fragment sizes and quantification. Equimolar amounts of the indexed samples were mixed and sequenced with Illumina MiSeq (Illumina Inc., San Diego, CA, United States) at the National Genomics Infrastructure/Science for Life Laboratory Stockholm.

After sequencing, nine samples with sequencing reads of less than 10,000 were excluded from further downstream analysis, resulting in a final dataset of 90 samples comprising 50 samples with periodontitis and 40 samples without periodontal disease (non-periodontitis). After the exclusion of low-depth libraries, the median depth of sequencing was 195,300 reads per sample [interquartile range (IQR): 146,700–218,600 reads].

### Bioinformatics analysis

The raw paired-end sequences obtained from the Illumina sequencing were first checked for base call quality. The base quality checking was performed using the FastQC tool (Andrews, 2015). The Phred score (Q20) was used as a base quality score threshold for the analysis. Adapters were trimmed using TrimGalore (v0.6.4)^1^, and primer sequences were removed with the help of the cutPrimers tool (Kechin et al., 2017). A rarefaction curve was generated to ensure sufficient sequencing depth in order to proceed with further downstream analysis (Supplementary Figure S1). The curves were generated by using R package phyloseq to plot the sequencing depth of the samples vs. the diversity indices, which showed that all the samples had sufficient sequencing depth to capture most of the microbial community, as the curves stabilized after 10,000× coverage.

### Amplicon sequence variants estimation, taxonomic classification, and statistical analysis

The pre-processed paired-end sequences were used for further downstream analysis using various bioinformatic tools. First, the pre-processed reads were analyzed using Quantitative Insights into Microbial Ecology version 2 (QIIME2). Amplicon sequence variants (ASVs) generated using QIIME2 were used for functional interpretation of the microbiota (Bolyen et al., 2019).

To visualize the abundance of taxonomy, sample-wise stacked bar plots were constructed at phylum, family, and genus levels using the *ggplot2* (3.2.1) R package. The results were further analyzed with the *phyloseq* (1.28.0) R package to study the alpha and beta diversity of the samples. Alpha diversity was calculated using the *estimate_richness* R function and visualized using the *ggplot2* R package. Beta diversity was estimated using the *ordinate* R function and visualized using the *plot_ordination* R function. The clustering of the samples was presented with a *non-metric multidimensional scaling* (NMDS) plot based on Bray–Curtis distance. Rarefaction analyses were conducted using the *rarefy* (*Vegan* v2.6-2) R function. Permutational multivariate analysis of variance (PERMANOVA) was performed to test for significant differences between the two groups at the genus taxonomic level using the *vegan* (version 2.4-3) R package. The analysis compared the groups and provided the top organisms that were responsible for their differentiation (Anderson, 2017). Statistical tests were performed between NMDS1 and NMDS2 to obtain the significant coordinate, and Welch’s *t*-test (two) was used to calculate the *p*-values. Correlation between microbial taxa and periodontal clinical parameters was assessed by the Spearman rank correlation coefficient (significance level *p* < 0.05) using the *psych* v2.2.3 R package. The graphical representation of the results was done using GraphPad Prism v8.4.2, where red indicates a positive correlation and blue indicates a negative correlation.

## Results

### Subgingival plaque microbiota composition and its relationship with periodontitis

The study cohort comprised 90 participants separated into two groups: the periodontitis group (*n* = 50) and the non-periodontitis group (*n* = 40). The mean age was 58.4 ± 2.7 years for the periodontitis group and 59.8 ± 2.9 years for the non-periodontitis group. We also categorized the patients based on their longitudinal follow-up of 10 years as having cancer (*n* = 35) at the time of sample collection (CSC, *n* = 13), those who developed cancer later (DCL, *n* = 22), and controls who did not have any cancer at the time of sampling but also did not develop cancer during the follow-up period (*n* = 55).

**Figure 1:**
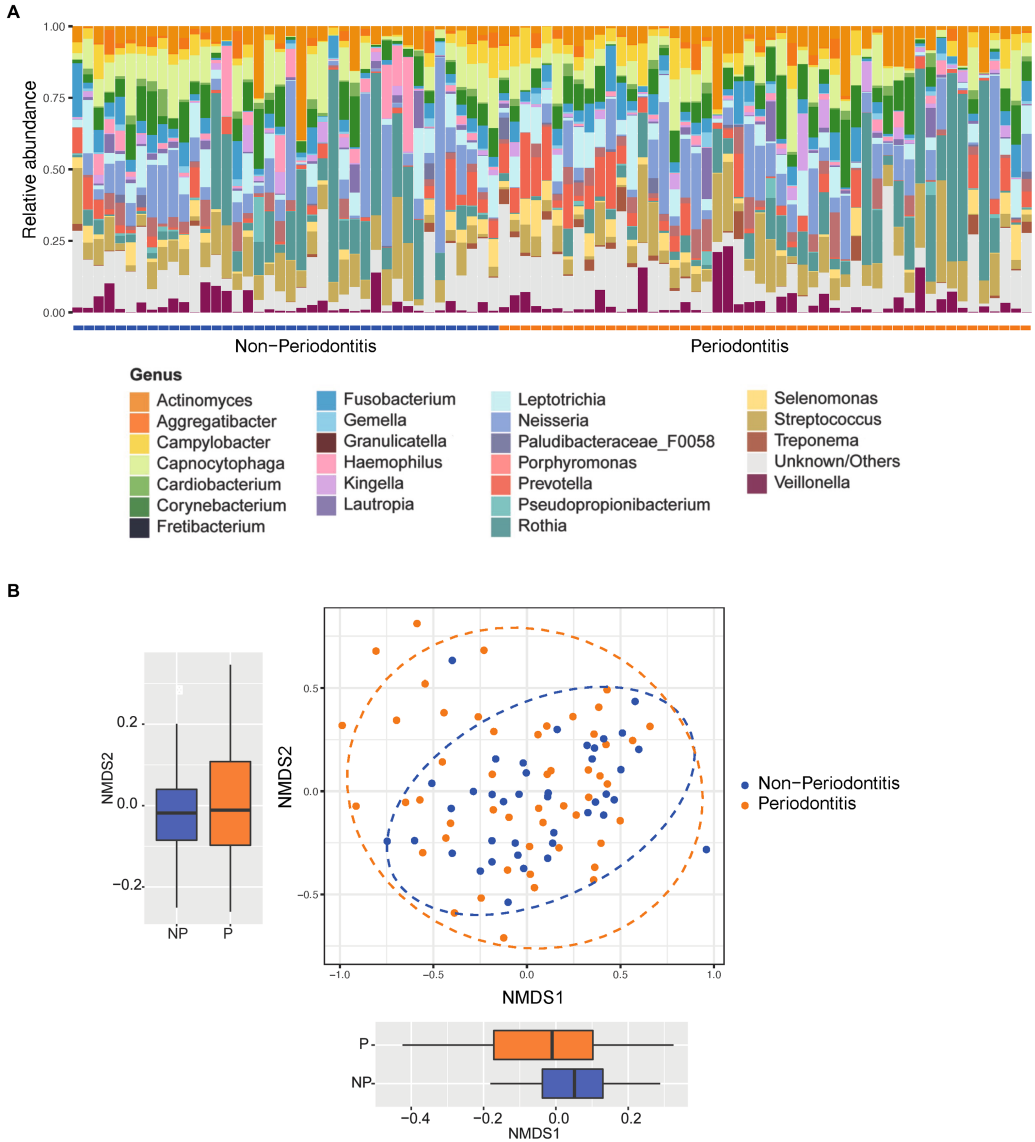
Relative abundance and beta diversity between groups. **(A)** Relative abundance of the most abundant organisms of all the 90 samples at the genus level grouped according to disease status (i.e., periodontitis or non-periodontitis). **(B)** Difference in beta diversity between the groups represented as non-metric multidimensional scaling (NMDS) ordination plots using Bray–Curtis distances; the separations between groups at each axis are seen in respective boxplot. The boxplots represent the median (horizontal black line), 25th and 75th quartiles (box edge), and upper and lower ends (whiskers).

Figure 1A shows the relative abundance distribution of all 90 included plaque samples at the genus level. The most prominent genera (phylum in brackets) across all samples were *Rothia*, *Corynebacterium*, *Actinomyces* (*Actinobacteria*), Neisseria (*Proteobacteria*), *Streptococcus* (*Firmicutes*), *Capnocytophaga*, *Prevotella* (*Bacteroidetes*), and *Leptotrichia* (*Fusobacteria*). Figure 1B shows the Beta diversity of samples visualized using a non-metric multidimensional scaling (NMDS) plot. There were no clear clusters for the periodontitis and non-periodontitis groups. Statistical analyses showed no significant differences for NMDS2 but did show significant differences with NMDS1 (*p* < 0.05) between the periodontitis and non-periodontitis groups (Figure 1B). At the genus level, the samples belonging to the periodontitis and non-periodontitis groups were ordered as per the NMDS1 ordinates to visualize the differences in the bacterial composition between each sample, as shown in Figure 1B. A boxplot of alpha diversity indices with corresponding *p*-values (Supplementary Figure S2) did not show any significant differences between groups.

**Figure 2:**
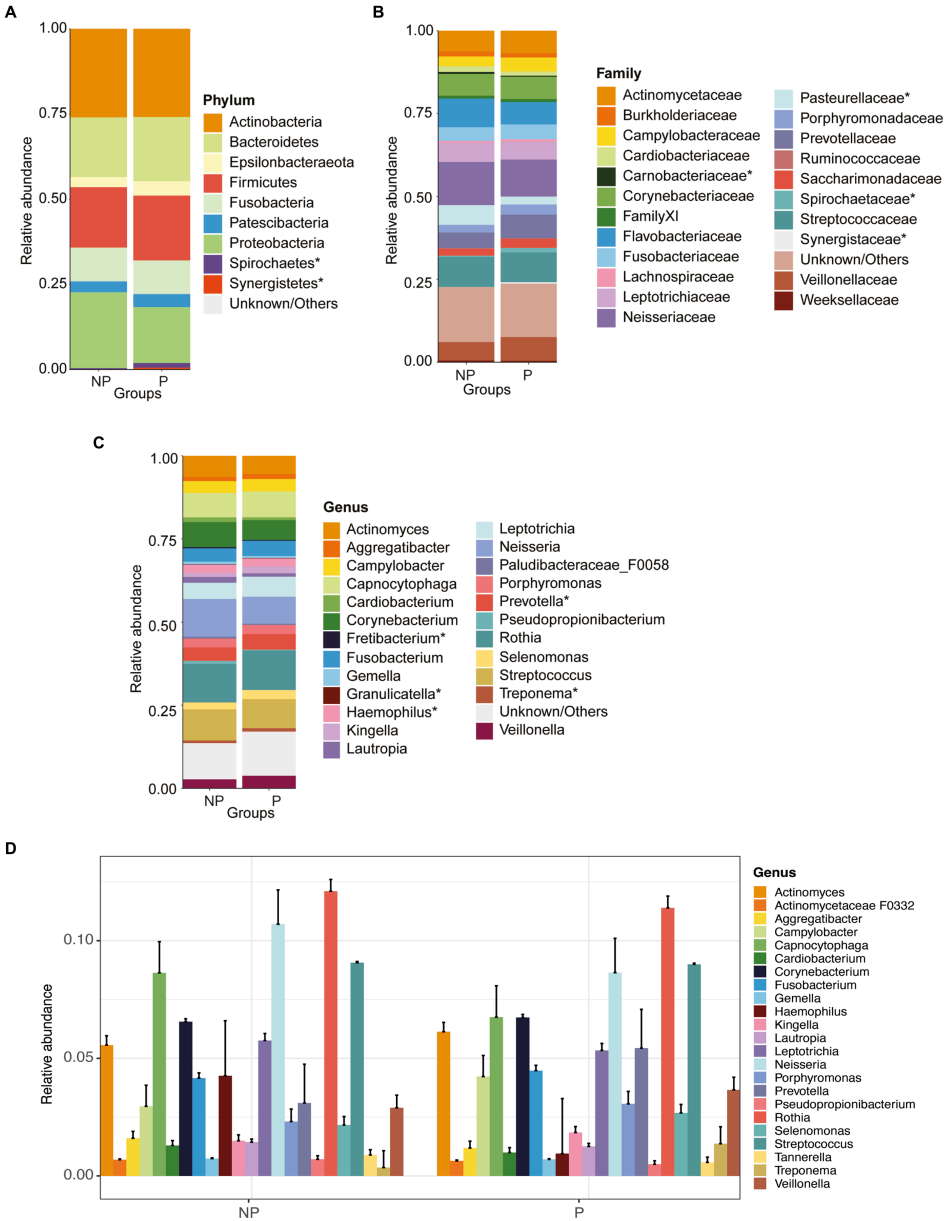
Relative abundance plots of microbiome compositions at different taxonomic levels and comparisons of alpha and beta diversity between periodontitis (P) and non-periodontitis (NP) individuals. Distribution of abundant organisms at the **(A)** phylum, **(B)** family, and **(C)** genus levels. **(D)** Extended error barplot showing the most abundant microbiome compositions between periodontitis and non-periodontitis individuals at the genus level. Organisms with a mean relative abundance of at least 1% across all samples are represented in different colors, whereas those with <1% abundance and unclassified are represented as “Unknown/Others”. The microbial communities denoted with an asterisk (*) are the significant ones between groups.

When comparing the periodontitis and non-periodontitis groups, the phyla *Firmicutes*, *Bacteroidetes* and *Epsilonbacteraeota* were found to be more abundant in individuals having periodontitis, whereas *Proteobacteria* and *Fusobacteria* were more abundant in the non-periodontitis group. The phylum *Actinobacteria* was found at similar levels in both the periodontitis and non-periodontitis groups (Figure 2A). The abundances of phyla *Firmicutes*, *Bacteroidetes*, *Proteobacteria Fusobacteria* and *Epsilonbacteraeota* were not significantly different between the groups. However, the abundances of *Spirochaetes* and *Synergistetes* were significantly different between the groups (*p* = 0.01 and *p* = 0.023, respectively).

At the family level, the most abundant bacteria were *Actinomycetaceae*, *Cardiobacteriaceae*, *Flavobacteriaceae*, *Neisseriaceae*, *Prevotellaceae*, *Veillonellaceae*, and *Streptococcaceae* (Figure 2B). The abundances of *Pasteurellaceae*, *Spirochaetaceae*, *Synergistaceae*, and *Carnobacteriaceae* were significantly differentially abundant between the two groups (*p* = 0.004, *p* = 0.01, *p* = 0.013, *p* = 0.023, and *p* = 0.032, respectively). The most abundant bacteria at the genus level were *Actinomyces*, *Corynebacterium*, *Neisseria*, *Prevotella*, *Streptococcus*, and *Rothia* (Figures 2C,D). When comparing the periodontitis vs. non-periodontitis samples, the abundances of *Haemophilus*, *Treponema*, *Fretibacterium*, *Granulicatella*, and *Prevotella* were significantly different between the groups (*p* = 0.01, *p* = 0.013, *p* = 0.023, *p* = 0.03, and *p* = 0.031, respectively) (Figure 2C). Of these, *Treponema*, *Fretibacterium*, and *Prevotella* were significantly more abundant in samples of periodontitis patients compared to non-periodontitis individuals.

**Figure 3:**
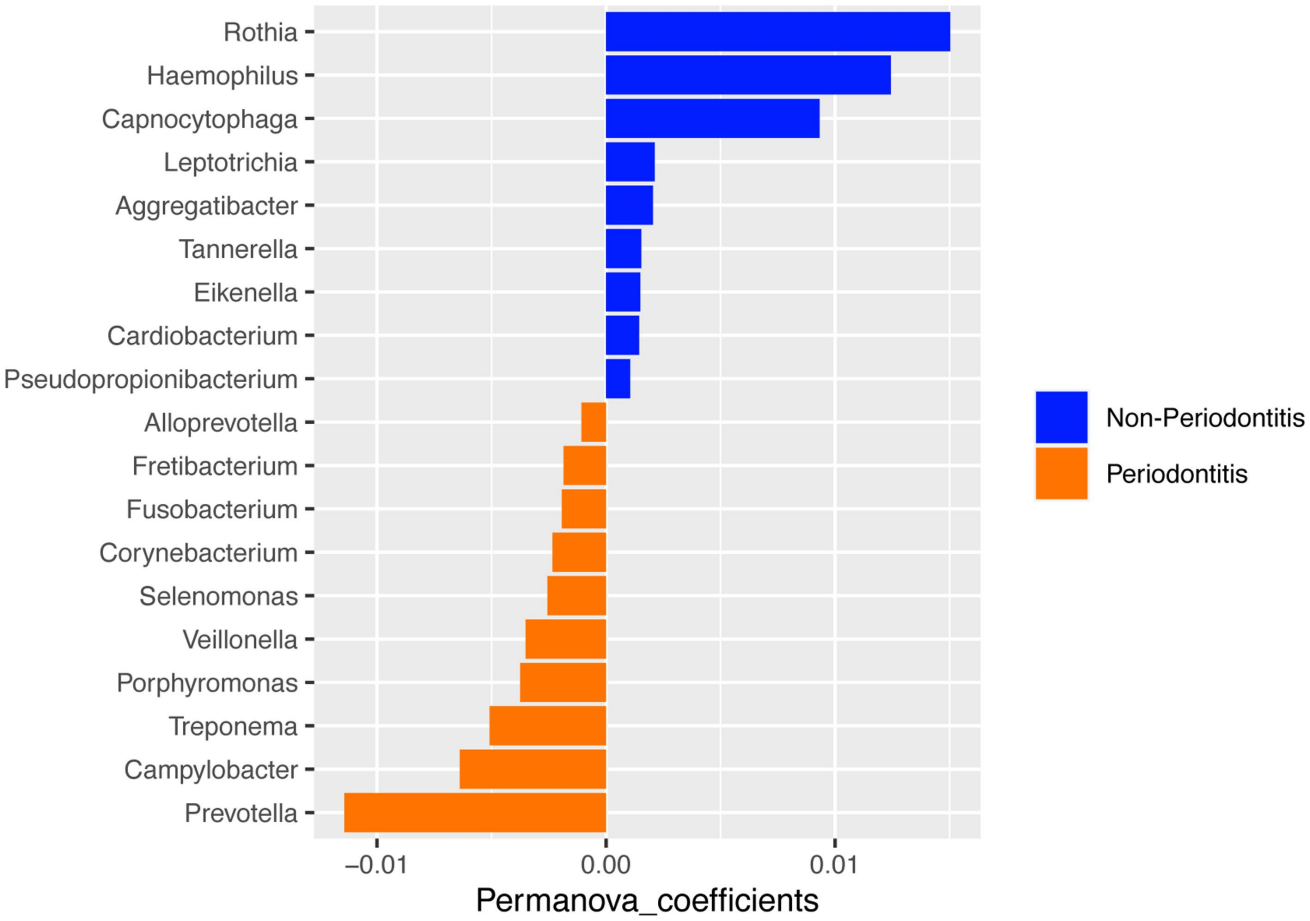
PERMANOVA analysis of microbial composition between the periodontitis and non-periodontitis groups at the genus level.

A PERMANOVA analysis was performed to test for differences between the two groups. When comparing the oral microbial composition between periodontitis and non-periodontitis individuals, at the genus level, the periodontitis microbiome had a high abundance of *Prevotella*, *Campylobacter*, and *Treponema*, whereas the non-periodontitis samples had *Rothia*, *Haemophilus*, and *Capnocytophaga* as the top-three most abundant genera (Figure 3). However, there were no significant differences in overall microbial composition between the two groups (*p* = 0.27).

### Subgingival microbiota composition and its association with cancer

**Figure 4:**
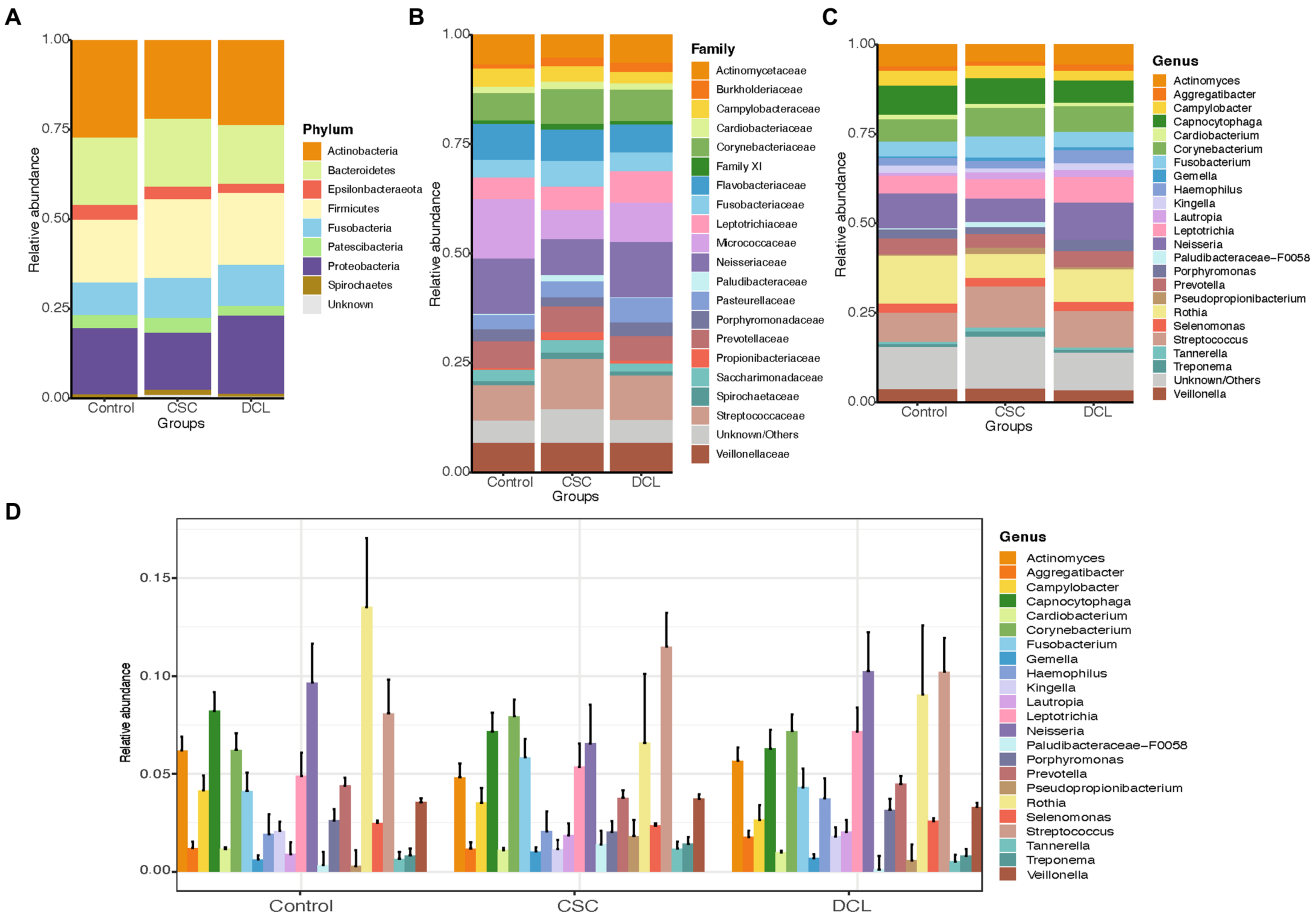
Distribution of the most abundant microbiome compositions between control, cancer at sample collection (CSC), and developed cancer later (DCL) groups at the **(A)** phylum, **(B)** family, and **(C)** genus levels. **(D)** Extended error barplot showing the distribution of the most abundant microbiome compositions between control, CSC and DCL groups at the genus level. Organisms with a mean relative abundance of at least 1% across all samples are represented in different colors, whereas those with <1% abundance and unclassified are represented as “Unknown/Others”.

Next, we categorized the patients included in this study based on their longitudinal follow-up of 10 years as having cancer (CSC), developed cancer later (DCL), and controls (Figure 4). The most abundant bacteria at the phylum level were *Actinobacteria*, *Proteobacteria*, *Firmicutes*, and *Bacteroidetes*. A comparison of the three groups revealed that *Actinobacteria* were more abundant in the non-cancer control group, as were *Firmicutes* in the CSC group, whereas *Proteobacteria* were enriched in the DCL group (Figure 4A). The abundant phyla *Firmicutes* and *Bacteroidetes* were not statistically significant between among all groups.

At the family level, S*treptococcaceae* and *Corynebacteriaceae* were enriched in the CSC group, whereas *Flavobacteriaceae*, *Micrococcaceae* and *Neisseriaceae* were more abundant in the control group, and *Leptotrichiaceae* were more highly abundant in the DCL group (Figure 4B). Moreover, *Paludibacteraceae* was found to be significantly more enriched in the CSC group compared to both the control and DCL groups (*p* = 0.019 and *p* = 0.02, respectively). At the genus level, *Streptococcus*, *Corynebacterium* and *Fusobacterium* were more abundant in the CSC group*; Neisseria*, *Rothia*, and *Capnocytophaga* were abundant in the control group; and *Prevotella* were more abundant in the DCL group (Figures 4C,D). The genus *Paludibacteraceae-F0058* was significantly (*p* = 0.02) enriched in the CSC group compared to both the controls and the DCL group.

**Figure 5:**
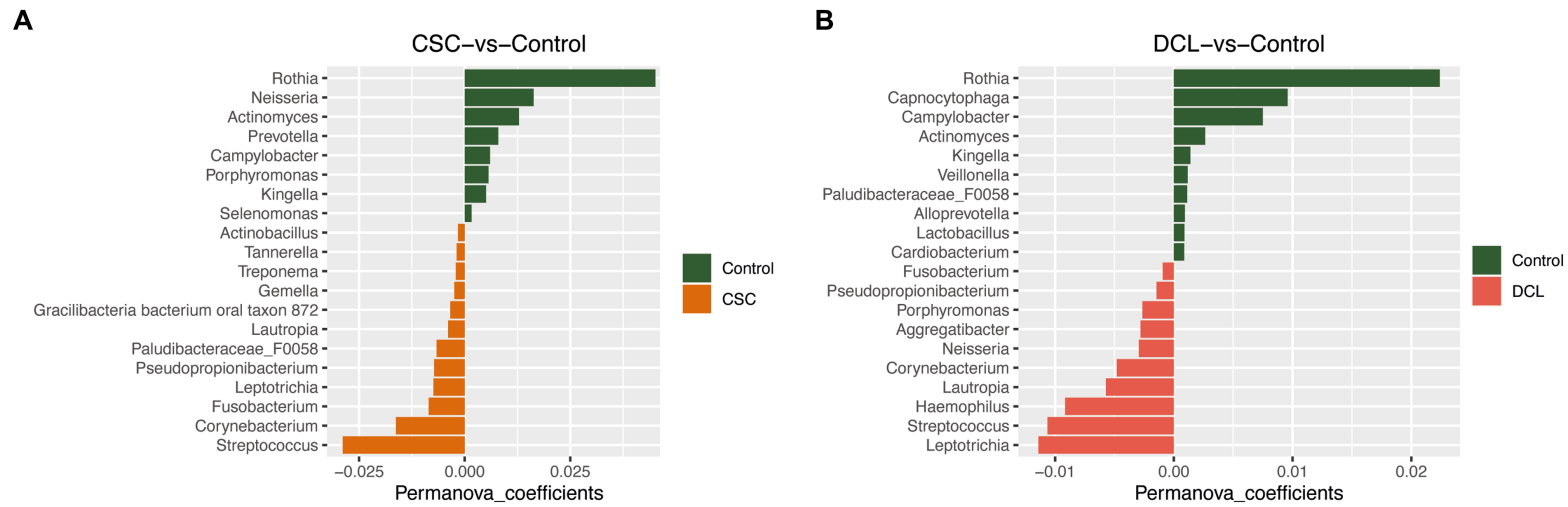
PERMANOVA analysis of microbial composition between control and cancer groups at the genus level: **(A)** cancer at sample collection (CSC) vs. control; **(B)** developed cancer later (DCL) vs. control.

A PERMANOVA analysis was performed to compare the subgingival microbiota between the two different cancer groups (CSC and DCL) and the control group. The results revealed that *Streptococcus*, *Corynebacterium*, and *Fusobacterium* were enriched in the CSC group, whereas *Rothia*, *Neisseria*, and *Actinomyces* were enriched in the control group (Figure 5A). Furthermore, *Leptotrichia*, *Streptococcus*, and *Haemophilus* were the top-three most abundant genera in the DCL group, whereas *Rothia*, *Capnocytophaga*, and *Campylobacter* were more abundant in the control group (Figure 5B). The PERMANOVA analysis was also used to compare the cancer group (CSC and DCL groups combined) with the control group (with no cancer diagnosis). The results showed that the phyla *Firmicutes*, *Fusobacteriota*, *Proteobacteria*, and *Spirochaetes* were more abundant in samples from patients diagnosed with cancer, whereas *Actinobacteria*, *Bacteroidetes*, *Epsilo bacteraeota*, and *Patescibacte* were more abundant in samples from the control group (Figure not shown).

We also analyzed the relationship between periodontitis and cancer using an NMDS plot, and no clear clustering was observed between four groups (Supplementary Figure S4), which were categorized based on having or not having periodontitis and cancer. The four groups were individuals with non-periodontitis with cancer, NPC (*n* = 15); individuals with both periodontitis and cancer, PC (*n* = 19); individuals with periodontitis but no cancer, PNC (*n* = 31); and individuals with non-periodontitis and without cancer, NPNC (*n* = 25). When comparing these groups, *Pseudopropionibacterium* was differentiated between PC and PNC; as was *Granulicatella*, *Lautropia*, *Haemophilus*, and *Desulfobulbus* between NPC and PNC; and *Eubacterium saphenum*, *Filifactor*, and *Desulfobulbus* between NPC and PC.

### Correlations between microbiota and periodontal clinical variables

**Figure 6:**
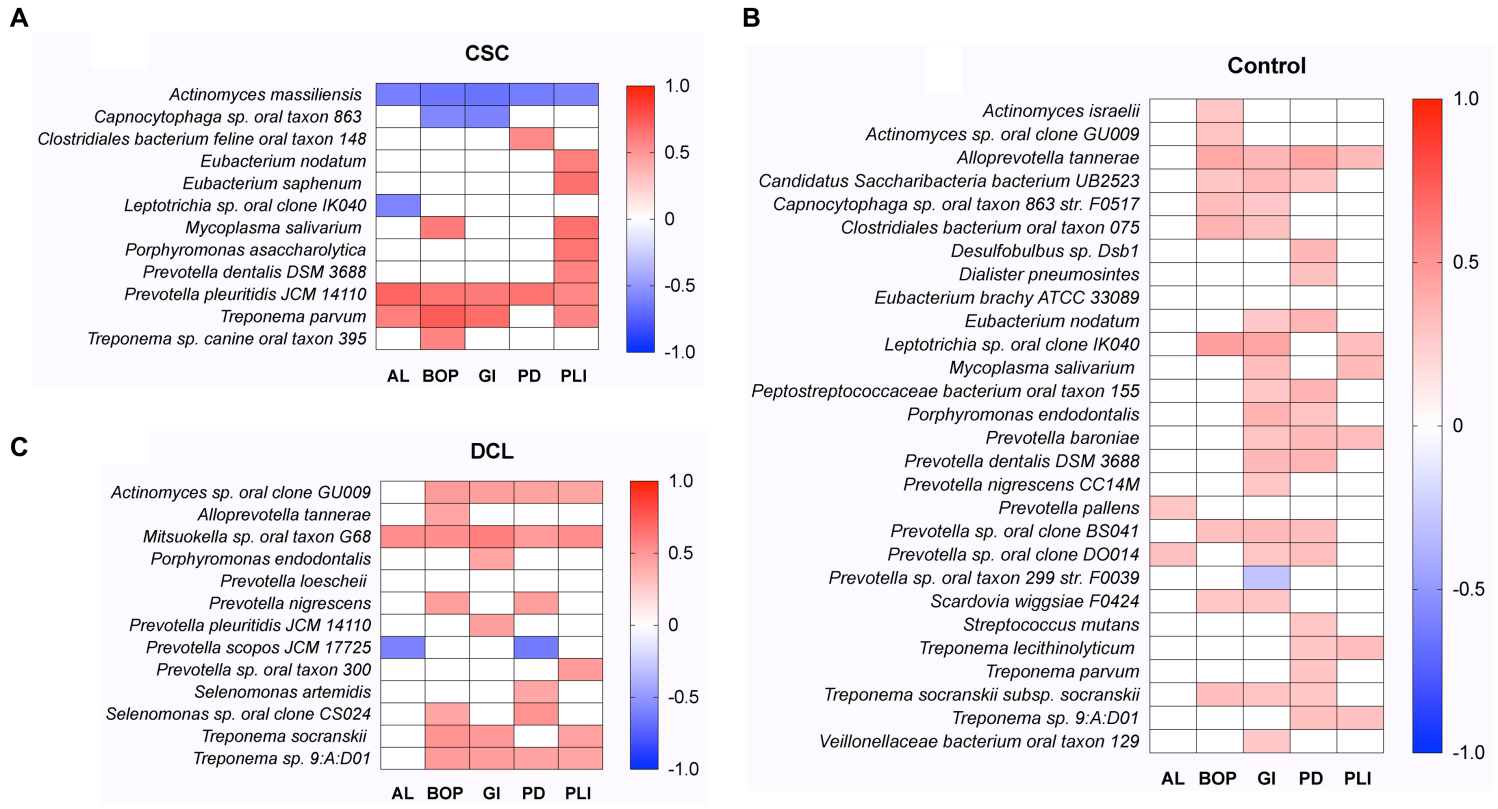
Spearman rank correlation analysis between subgingival microbiota and periodontal clinical parameters (AL, BOP, GI, PD, PLI). The heatmap shows statistically significant (*p* < 0.05) correlations between microbial taxa (at the species level) and periodontal parameters for the **(A)** cancer at sample collection (CSC), **(B)** developed cancer later (DCL), and **(C)** control groups. Positive correlations are displayed in red and negative correlations in blue color.

A correlation analysis (for *p* < 0.05) was also performed to investigate the relationship between microbiota and periodontal disease. A matrix of the correlations between the periodontal clinical parameters AL, BOP, GI, PD, and PLI and microbial taxa (species level) for the three different groups, CSC, DCL and controls, is illustrated in Figure 6. In the CSC group, strong positive correlations were observed between the periodontal parameters AL, BOP, and GI and the species *Prevotella pleuritidis* and *Treponema parvum* (coefficients ranging from 0.6 to 0.75). In addition, PLI strongly correlated with the bacteria *Eubacterium nodatum*, *Eubacterium saphenum*, *Mycoplasma salivarium*, *Porphyromonas asaccharolytica*, *Prevotella dentalis*, *Prevotella pleuritidis*, and *Treponema parvum*. Negative correlations were found between AL, BOP, GI, PD, and PLI and *Actinomyces massiliensis;* as well as between *Capnocytophaga* sp. *oral taxon* and BOP and GI values (Figure 6A). In the DCL group, the strongest correlations (coefficients ranging from 0.50 to 0.59) were shown between all the periodontal parameters and the bacterium *Mitsuokella sp. oral taxon*. In this group, *Prevotella scopos JCM* was negatively correlated with AL and PD scores (Figure 6B). In contrast to the two cancer groups, CSC and DCL, no strong correlations (ranging between 0.27 to 0.45) were observed in the control group between the periodontal variables and the significantly abundant species (Figure 6C).

## Discussion

Periodontal infection causes chronic inflammation in the oral cavity and is considered an important statistical risk factor for several types of cancer (Hajishengallis, 2014; Hajishengallis, 2015; Flemer et al., 2018). Some have proposed that the association between periodontitis and the risk of different types of cancer is due to the chronic inflammation caused by periodontitis, which drives cancer development by infiltration of leukocytes in the tumor microenvironment (Hanke et al., 1990; Garrett, 2015). Numerous studies have indeed reported a relationship between periodontal disease and various types of cancer (Aas et al., 2005; Keijser et al., 2008; Bik et al., 2010; Segata et al., 2011; Soder et al., 2011, 2021; Norder Grusell et al., 2013; Dong et al., 2018; Bai et al., 2022). In addition, studies have also shown that the oral microbiota may contribute to carcinogenesis by altering the homeostasis/cellular metabolism, the immune responses creating a proinflammatory microenvironment, cell migration and production of carcinogenic metabolites (Garrett, 2015; Bai et al., 2022; Lamont et al., 2022). In the current study, we aimed to investigate the subgingival microbial composition in periodontal health and disease and its relationship with the diagnosis and development of cancer in a longitudinal 10-year follow-up study. Our 16S rRNA results identified several significant genera differentiating individuals with periodontitis from those without periodontitis (*Haemophilus*, *Treponema*, *Fretibacterium*, *Granulicatella, Prevotella and Defluviitaleaceae UCG-011*). However, only one genus (*Pseudopropionibacterium*) appeared to be significantly different between the cancer patient samples and controls with no cancer.

Our bacterial abundance study was carried out at different taxonomic levels, including phylum, family, and genus. First, we analyzed the subgingival microbial composition in periodontally healthy individuals and patients with periodontitis. In agreement with previous findings, the most abundant bacteria at the phylum level were *Actinobacteria*, *Proteobacteria*, *Firmicutes,* and *Bacteroidetes* (Griffen et al., 2012; Cai et al., 2021; Sedghi et al., 2021). *Firmicutes* and *Bacteroidetes* were more abundant in the periodontitis group compared to the non-periodontitis group, also confirming previous findings reporting an increased abundance of *Bacteroidetes* and *Firmicutes* in periodontitis (Segata et al., 2012; Sedghi et al., 2021). The most abundant microbial communities at the genus level were *Actinomyces*, *Corynebacterium*, *Neisseria*, *Prevotella*, *Streptococcus,* and *Rothia*, whereas *Haemophilus*, *Treponema*, *Fretibacterium*, *Granulicatella*, *Prevotella*, and *Defluviitaleaceae UCG-011* genera were significantly different when comparing individuals with and without periodontitis. Notably, samples from periodontitis patients had significantly higher levels of *Treponema*, *Fretibacterium*, and *Prevotella* compared to non-periodontitis individuals. Thus, our results confirm earlier research as *Treponema*, *Fretibacterium*, and *Prevotella* (belonging to the phyla *Spirochetes*, *Synergistetes*, and *Bacteroidetes*, respectively) are reported to play essential roles in the pathogenesis of periodontitis (Perez-Chaparro et al., 2014; Hajishengallis, 2015; Lundmark et al., 2019). Similarly, the genus *Defluviitaleaceae UCG-011* (belonging to phylum *Firmicutes*) is more abundant in supragingival plaque samples of periodontitis than healthy controls (Kawamoto et al., 2021). *Treponema*, on the other hand, is a diverse bacterial genus and a constituent of healthy oral flora; however, with a vital role in the etiology and pathogenesis of periodontal disease, its reduction prompts the dysbiosis of microbiota (Buyuktimkin et al., 2019; Velusamy et al., 2019; Li et al., 2021).

Second, we analyzed the distribution of subgingival microbial composition and its relationship with occurrence of cancer in the CSC and DCL groups. At the phylum level, similar microbial composition was observed between the cancer and control groups. Indeed, *Actinomyces*, *Corynebacterium*, *Fusobacterium*, *Neisseria*, *Prevotella*, *Rothia*, and *Streptococcus* were the differentially abundant genera found in the periodontitis as well as in the cancer groups. In agreement with our findings, microbes including *Fusobacterium*, *Streptococcus*, and *Prevotella* have been detected in high abundance in cancerous periodontal tissues (Dong et al., 2018). Consistent with these findings, genomic analysis, 16S rDNA sequence analysis, and quantitative PCR have revealed that *Fusobacterium* sequences are enriched in colorectal carcinoma (Kostic et al., 2012). When comparing the three groups (CSC, DCL and controls) at the genus level, *Neisseria*, *Rothia*, and *Capnocytophaga* were more abundant in the control group, whereas *Corynebacterium* and *Streptococcus* were more abundant in the CSC group, and *Prevotella* were more abundant in the DCL group. Indeed, the genus *Corynebacterium*, has been shown to be enriched in saliva samples of gastric cancer patients (Oliveira et al., 2017). Similarly, strains of *Streptococcus* have been reported to be involved in numerous types of cancer including colorectal adenocarcinomas and gastric cancer (Abdulamir et al., 2011). On the other hand, a higher abundance of *Corynebacterium* has also been associated with reduced risk of head and neck squamous cell cancer, as well as good oral health (Meuric et al., 2017; Hayes et al., 2018).

Several subgingival genera were differentially enriched among the study groups. For example, *Pseudopropionibacterium* (phylum *Actinobacteria*) was found to be significantly enriched in the periodontitis group with cancer, whereas *Eubacterium saphenum*, *Filifactor*, and *Desulfobulbus* were found to be enriched in the non-periodontitis group with cancer, suggesting that these genera may be involved in cancer development. Notably, *Pseudopropionibacterium* has been found to be more prevalent in cases of apical periodontitis and to have a significant difference in abundance in esophageal cancer cases compared to controls (Liu et al., 2020; Perez-Carrasco et al., 2023).

Our PERMANOVA results showed that *Rothia* were the most prevalent genus in the oral microbiota in both the non-cancer and non-periodontitis groups, which suggests that this genus may have a protective effect on periodontitis and cancer development. This hypothesis is supported by a previous study demonstrating that the genus *Rothia* were more prevalent in healthy controls compared to subjects with oral squamous cell carcinoma (Zhao et al., 2017).

Finally, we investigated the inter-relationship and correlations between the microbial communities and periodontitis/periodontal parameters and cancer (in the CSC and DCL samples). The differential abundance of microbial composition was confirmed using a PERMANOVA analysis, which compared the bacterial compositions between two groups and characterized the top discriminative taxa between them. The alpha diversity results showed that non-periodontitis individuals had lower diversity compared to periodontitis patients. However, there were no significant differences in the diversity between periodontitis and non-periodontitis groups (Supplementary Figure S2). Similarly, there were no significant differences in the diversity between the CSC, DCL, and control groups (Supplementary Figure S3). Notably, the correlation analysis, which was performed to evaluate the relationship between microbiota and different clinical periodontal parameters, revealed strong correlations between BOP, GI, PLI, and several species belonging to genera *Prevotella*, *Treponema*, and *Mycoplasma* in the CSC group. Both BOP and GI are well-known indicators of gingival inflammation, which may thus contribute to the development of cancer.

The strength of our study was the homogeneity of the subject material, as the cohort (*n* = 1,676) was followed-up together for over 30 years, with cumulated disease data from the national population registers of Sweden. The current study is a clinically examined sample of the large cohort that was followed-up for 10 years. However, the study was limited by the relatively small 10-year sample that was available for the present investigation, as well as the relatively short follow-up period considering the development of new cancer cases. Indeed, the development of cancer is a slow process. With regard to the cross-sectional design of the study, providing a snapshot of the microbiota collected at one point in time, longitudinal studies that follow participants over time are needed to gain deeper insights into the relationship between oral microbiota and various types of cancer. Another limitation is that the study did not consider confounding factors such as smoking, medication use or diet into consideration, which may have influenced the composition of the subgingival microbiota. Furthetmore, in the current study, we did not differentiate between healthy and diseased sites when pooling subgingival samples from each participant, which may have contributed to the lack of clear beta diversity clusters between the periodontitis and non-periodontitis groups. This is consistent with previous findings indicating that the subgingival microbiota can differ between healthy and diseased sites in patients with periodontitis, suggesting a site-specific presence of periodontal pathogens in plaque samples (Belstrøm et al., 2017).

In conclusion, in the present study, we identified several genera differentiating periodontitis from non-periodontitis groups of subjects (*Haemophilus*, *Treponema*, *Fretibacterium*, *Granulicatella, Prevotella* and *Defluviitaleaceae UCG-011*). Only one genus, *Pseudopropionibacterium*, differentiated individuals with periodontitis having cancer (PC) from periodontitis patients without any cancer (PNC). Additionally, in the CSC group, we also found that the presence of periodontal inflammation (as reflected in BOP, GI, and PLI scores) strongly correlated with species belonging to the genera *Prevotella*, *Treponema*, and *Mycoplasma*. Collectively, our findings revealed significant differences in the subgingival microbiota among the studied groups, underscoring the need for further investigation into the potential role of oral pathogens in the development of cancer.

## Data availability statement

The original contributions presented in the study are publicly available. This data can be found at: NCBI BioProject, accession number: PRJNA985445 [https://www.ncbi.nlm.nih.gov/bioproject/985445].

## Ethics statement

The studies involving human participants were reviewed and approved by Ethics Committee of the Karolinska University Hospital at Huddinge (Dnr 2007/1669-31; 2012/590-32; 2017/2204-32). Written informed consent for participation was not required for this study in accordance with the national legislation and the institutional requirements.

## Author contributions

BS, TY-L, JM, and UN contributed to the conception and design of the study. BS contributed to the clinical dental examinations and sample collection. AL and YH performed all the experiments. AN and UN performed the bioinformatical and statistical analyses. TY-L, AN, and UN wrote the first draft of the manuscript. All authors contributed to the article and approved the submitted version.

## Conflict of interest

The authors declare that the research was conducted in the absence of any commercial or financial relationships that could be construed as a potential conflict of interest.

## Publisher’s note

All claims expressed in this article are solely those of the authors and do not necessarily represent those of their affiliated organizations, or those of the publisher, the editors and the reviewers. Any product that may be evaluated in this article, or claim that may be made by its manufacturer, is not guaranteed or endorsed by the publisher.

## Footnotes

^1^https://www.bioinformatics.babraham.ac.uk/projects/trim_galore/

## Supplementary material

SUPPLEMENTARY FIGURE S1 Rarefaction curves generated according to the different alpha diversity indices between periodontitis and non-periodontitis individuals, aiming to determine how many microbial communities can be detected with increasing numbers of sequencing reads. The curves do not converge, indicating that increasing the number of sequencing depths may result in the identification of additional species.

SUPPLEMENTARY FIGURE S2 Boxplots comparing alpha diversity indices (Observed, Chao1, ACE, Shannon, Simpson, and Inverse simpson) between the periodontitis and non-periodontitis groups.

SUPPLEMENTARY FIGURE S3 Boxplots comparing alpha diversity indices (Observed, Chao1, ACE, Shannon, Simpson, and Inverse simpson) between the control, cancer at sample collection (CSC), and developed cancer later (DCL) groups.

SUPPLEMENTARY FIGURE S4 Relative abundance and beta diversity between non-periodontitis and cancer (NPC), periodontitis and cancer (PC), periodontitis and no cancer (PNC), and non-periodontitis and no cancer (NPNC) groups. The significant bacteria (*P*<0.05) at the genus level, comparing the different groups with the periodontitis vs. cancer groups, is presented.
