# Supplementary figures and images for: Composition of subgingival microbiota associated with periodontitis and diagnosis of malignancy—a cross-sectional study
Source: Front Microbiol. 2023 May 22;14:1172340. doi: 10.3389/fmicb.2023.1172340 (PMC10325785; doi:10.3389/fmicb.2023.1172340)

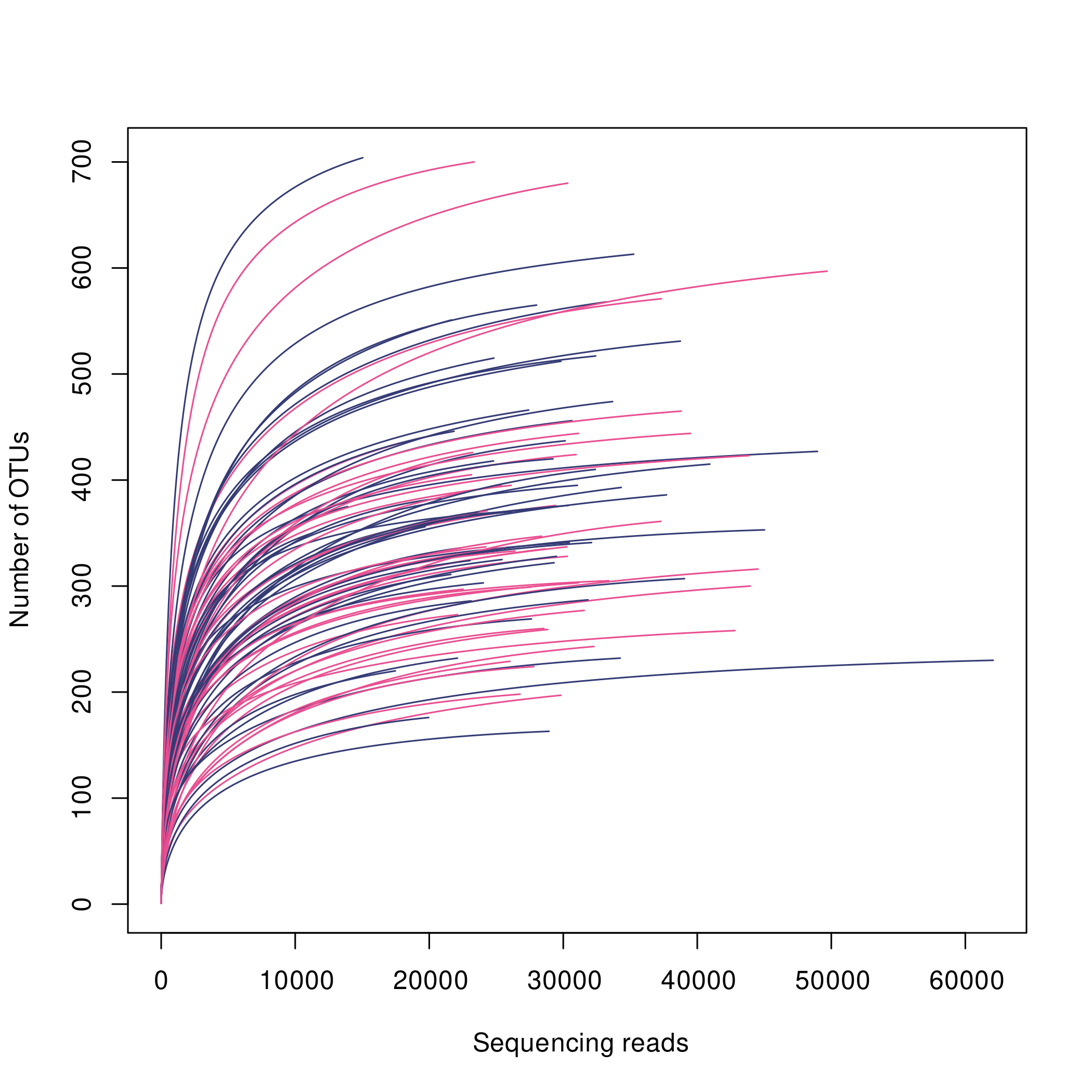

Supplement: SUPPLEMENTARY FIGURE S1 — Rarefaction curves generated according to the different alpha diversity indices between periodontitis and non-periodontitis individuals, aiming to determine how many microbial communities can be detected with increasing numbers of sequencing reads. The curves do not converge, indicating that increasing the number of sequencing depths may result in the identification of additional species. [file Image_1.TIFF]

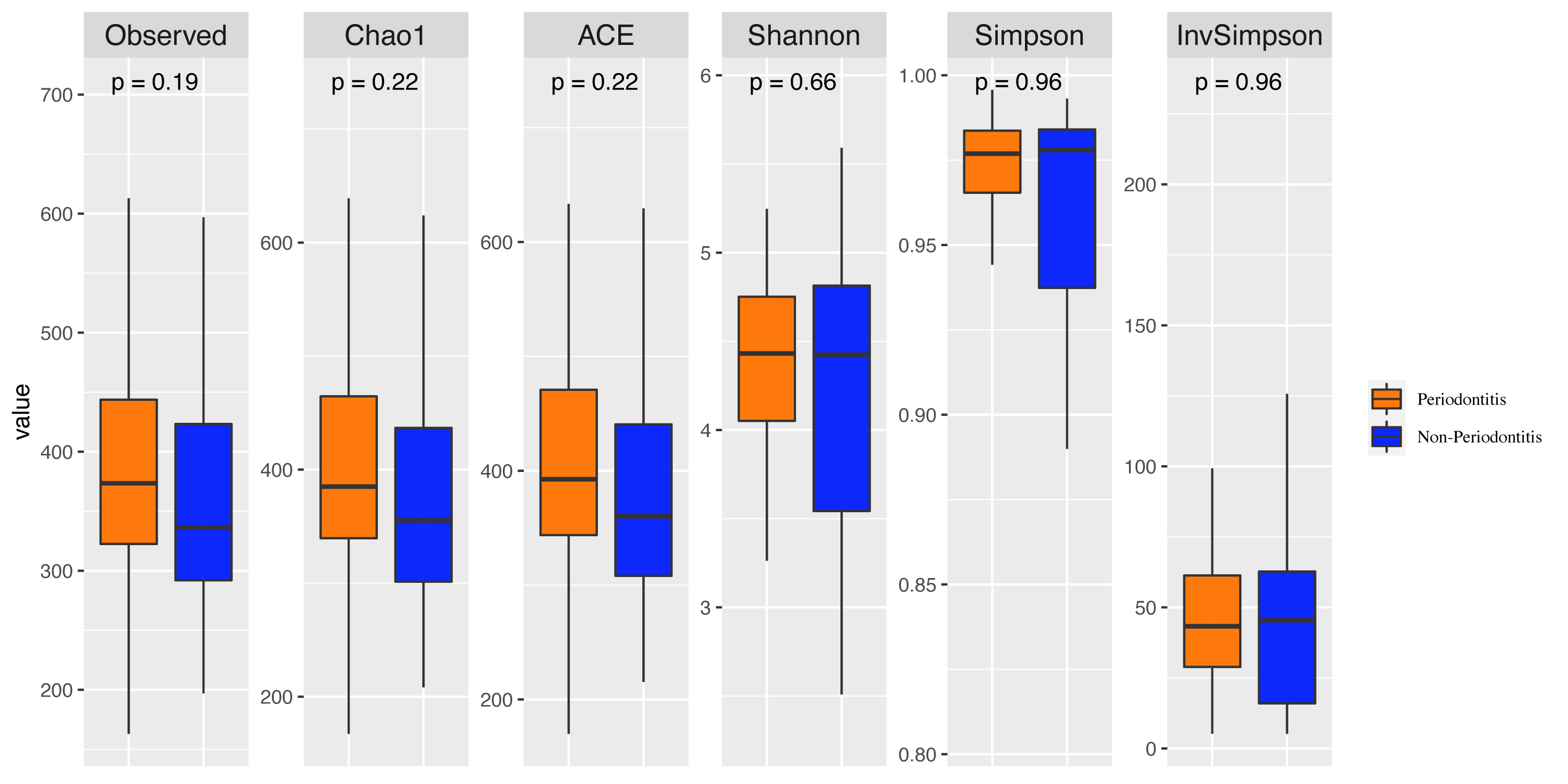

Supplement: SUPPLEMENTARY FIGURE S2 — Boxplots comparing alpha diversity indices (Observed, Chao1, ACE, Shannon, Simpson, and Inverse simpson) between the periodontitis and non-periodontitis groups. [file Image_2.TIFF]

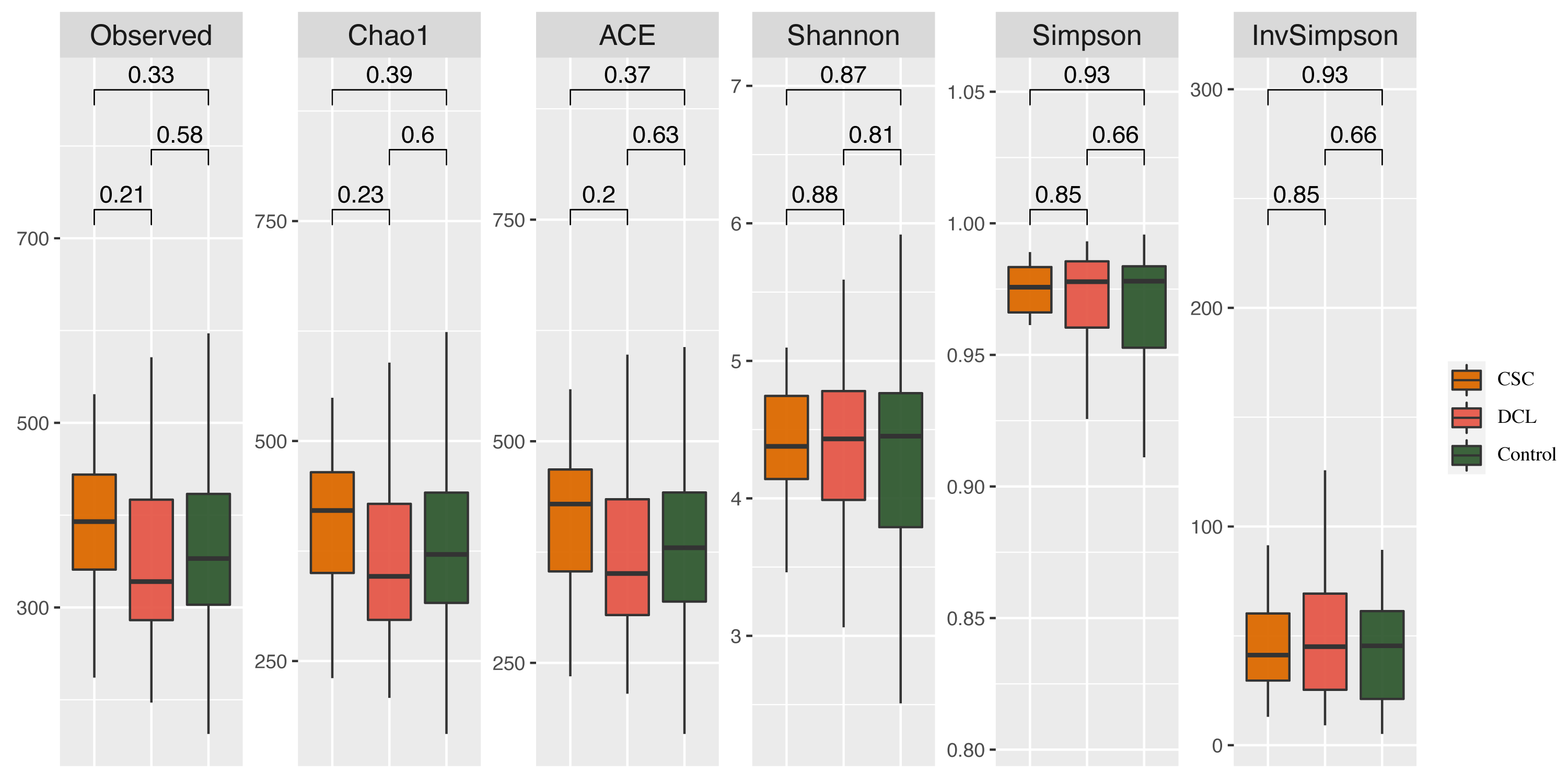

Supplement: SUPPLEMENTARY FIGURE S3 — Boxplots comparing alpha diversity indices (Observed, Chao1, ACE, Shannon, Simpson, and Inverse simpson) between the control, cancer at sample collection (CSC), and developed cancer later (DCL) groups. [file Image_3.TIFF]

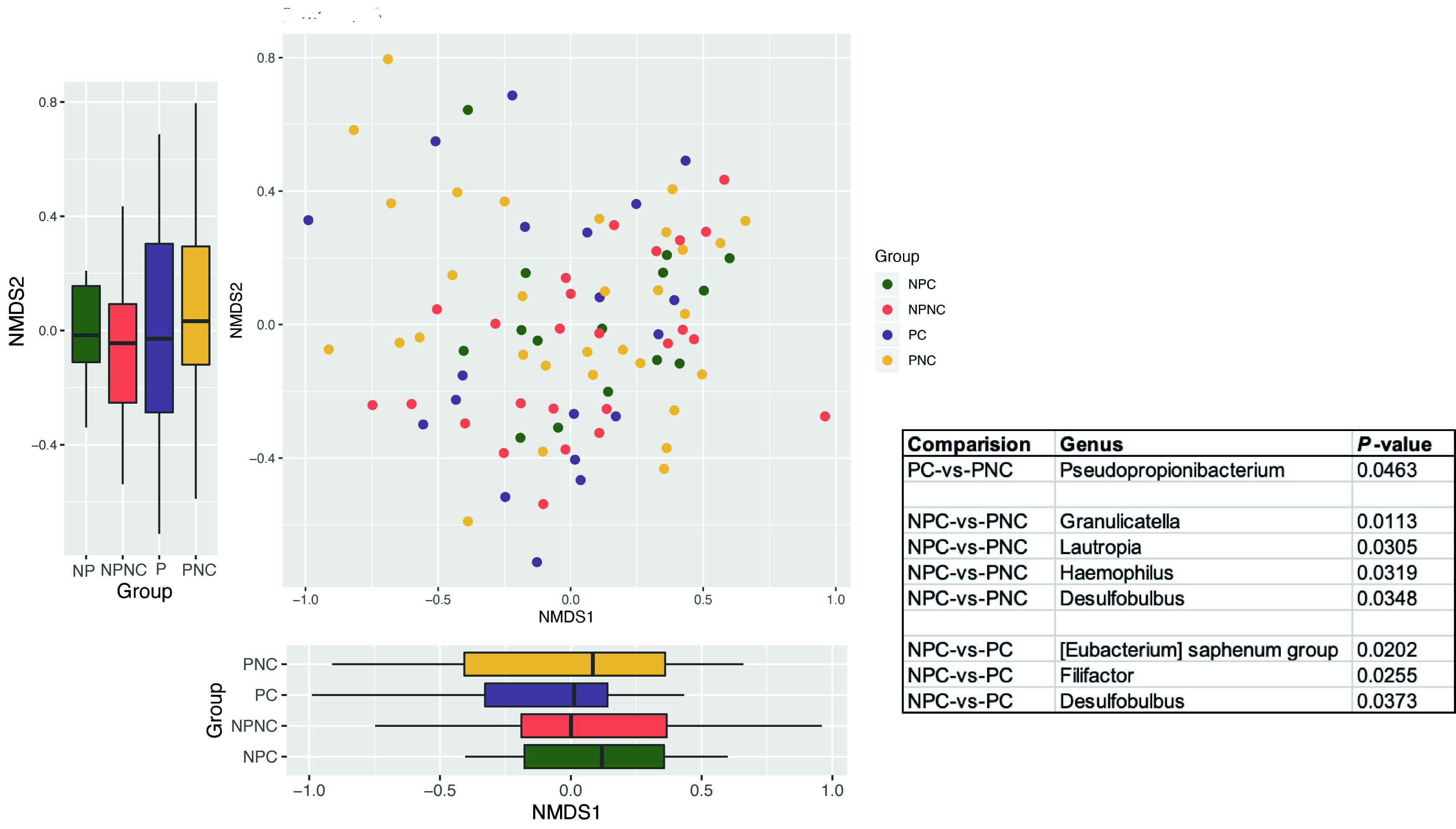

Supplement: SUPPLEMENTARY FIGURE S4 — Relative abundance and beta diversity between non-periodontitis and cancer (NPC), periodontitis and cancer (PC), periodontitis and no cancer (PNC), and non-periodontitis and no cancer (NPNC) groups. The significant bacteria (P<0.05) at the genus level, comparing the different groups with the periodontitis vs. cancer groups, is presented. [file Image_4.TIFF]
